# Digital Subtraction Angiography-Guided Foam Sclerotherapy with Polidocanol for Treating Superficial Venous Malformation

**DOI:** 10.3400/avd.oa.20-00164

**Published:** 2021-09-25

**Authors:** Tuan Tran Anh, Quyen Le Nguyen, Quynh Mai Thi, Thong Pham Minh

**Affiliations:** 1 Radiology Center, Bach Mai Hospital, Ha Noi, Vietnam; 2 Radiology Department, Hanoi Medical University, Ha Noi, Vietnam; 3 Department of Thoracic, Vascular and Neurology, Trung Vuong Hospital, Ho Chi Minh City, Vietnam

**Keywords:** digital subtraction angiography, sclerotherapy, venous malformation

## Abstract

**Objective:** This study aims to describe the angiographic imaging characteristics of superficial venous malformations and evaluate the treatment effectiveness of digital subtraction angiography (DSA)-guided foam sclerotherapy with polidocanol.

**Materials and Methods**: This prospective study was conducted in 18 patients with venous malformation treated by DSA-guided sclerotherapy. Treatment outcomes were evaluated based on pain improvement and reduction in lesion size on magnetic resonance imaging (MRI) 6-months posttreatment.

**Results:** A total 21 lesions and 46 sclerotherapy sessions were analyzed. MRI findings presented 8/21 lesions (38.1%) with excellent response, 9/21 (42.9%) with good response, and 3/21 (14.3%) with average response, while one patient (4.8%) showed no response. All patients experienced pain. A significant reduction (p<0.01) was observed in the pre- to posttreatment pain score (5.45 and 0.64, respectively). Over a follow-up period of 1–4 years, three out of four patients (75%) in the retrospective cohort experienced recurrence; one patient had an increased lesion size and pain score, while the other two patients only showed an increased lesion size. No severe complications were seen.

**Conclusion:** DSA-guided sclerotherapy with polidocanol is a safe and effective procedure for reducing lesion size and pain in symptomatic patients with superficial venous malformations.

## Introduction

Venous malformations are common vascular anomalies defined by low-flow and abnormal veins lacking valves.^1)^ Venous malformations result from an abnormal growth during the fetal period, and symptoms depend on the lesion size and location. The skin color of the lesions is determined by the depth of the lesions and ranges from colorless to purple. The progression of venous malformations usually involves a steady and slow increase in size over time. The morphology and size of venous malformations change with posture, and they can compress nearby organs, causing pain and limited movement. Venous malformations occur anywhere in the body but more commonly in soft tissues. Over the past decade, vein specialists globally have supported the use of ultrasound and magnetic resonance imaging (MRI) for the classification and diagnosis of venous malformations. While its indication and treatment effectiveness remain controversial, sclerotherapy is considered to be the most important treatment for venous malformations. Indications for treatment include the occurrence of symptoms affecting patient function and aesthetics.^2,3)^

Sclerotherapy is considered the first-line treatment for venous malformations.^1,4)^ Although various types of sclerosants exist, the most common is absolute alcohol. Ethibloc (a mixture of alcohol and zein) is a low-cost and effective sclerosing agent. Polidocanol (Aetoxisclerol) is a surfactant with a mechanism of action similar to that of absolute alcohol but with lesser toxicity and associated pain.^5,6)^ The effectiveness of sclerotherapy is assessed based on the lesion size and the symptoms for which treatment is indicated. Treatment success rates vary among studies. A Cochrane library meta-analysis revealed numerous flaws in studies investigating treatment effectiveness and indicated that there was insufficient evidence to accurately determine the optimum treatment method.^7)^ However, many researchers believe that sclerotherapy should be the first-line treatment due to its effectiveness, safety, and low cost.

Sclerotherapy-associated local complications such as necrotic ulceration of the skin or nerve damage have been reported; however, since polidocanol (Lauromacron 400) became the main sclerosant administered in the procedure, venous malformations are treated more frequently with reduced complications.^3)^ With the development of a classification for venous malformations by Puig et al. in 2003,^8)^ there has been an increasing number of studies evaluating the effectiveness of sclerotherapy under digital subtraction angiography (DSA) guidance worldwide. However, to the best of our knowledge, there have been no reports of this treatment method in Vietnam. Therefore, this study aimed to describe the angiographic features of superficial venous malformations and evaluate the treatment effectiveness of DSA-guided sclerotherapy with polidocanol in Vietnam.

## Materials and Methods

This study was approved by the Ethical Committee of Bach Mai Hospital under the reference number 08/BMH. Between 2015 and 2020, 18 patients were treated with venous malformations using DSA-guided sclerotherapy at the Bach Mai Radiology Center, Hanoi, Vietnam.

All patients were hospitalized because of clinical symptoms, such as swelling, pain, limited movement, aesthetics, dysphagia, and shortness of breath, and were diagnosed by ultrasound with a high-frequency flat probe (7–15 MHz). MRI contrast agents were administered to assess the size and extent of lesions. We inserted 18–22 G needles directly through the skin into the lesion. The contrast agent was injected with a short wire, and an angiogram was then performed. The morphology of the venous malformations and drainage veins were carefully observed on MRI by two vascular interventional radiologists and classified according to Puig’s classification: type I, isolated malformation without drainage vein; type II, malformation that drains into normal veins; type III, malformation that drains into dysplastic veins; and type IV, venous ectasia.^8)^

The foam sclerosant drug, polidocanol (4-ml polidocanol/time/person), was mixed according to the Tessari technique using tripods and two syringes and was then pumped into the venous malformation. The ratio of the gas and liquid sclerosant volume varies according to lesion features.^7)^ We mixed polidocanol, air, and contrast (in some cases) and determined this ratio based on the feature of lesions (e.g., flow, size of venous drainage, volume of lesion). If the lesions were too big and the patient agreed, we used over 4-ml polidocanol, because it was less costly. In our study, there was only one patient using over 4-ml polidocanol/time.

After the sclerotherapy, patients were followed up for at least 2 h and discharged on the same day. They were followed up for late complications, and evaluation of treatment success was conducted at 1 month, 3 months, and 6 months posttreatment.

Success of sclerotherapy treatment was assessed according to the following parameters: (1) lesion size on MRI (pre- and posttreatment), (2) pain score (visual analog scale [VAS]) pre- and posttreatment, and (3) recurrence: increased lesion size on MRI or increased VAS pain score.

SPSS v.20.0 software was used to statistically analyze and describe the patient characteristics, including age, lesion location, symptoms, and number of injections required for sclerotherapy. Follow-up treatment success was evaluated based on the reduction in lesion size (excellent, >90%; good, 50%–90%; average, 10%–50%; no response, <10%) and improvement in pain score. These were calculated by a descriptive statistical algorithm.

## Results

A total of 18 patients (7 males and 11 females) with venous malformations were enrolled. Their mean age was 26.5±12.9 years (range: 6–59 years). **Table 1** presents the patient characteristics. Venous malformations occurred in the head and neck in eight (38.1%) patients, lower extremities in nine (42.9%) patients, upper extremities in two (9.5%) patients, and trunk in two (9.5%) patients. The majority of lesions were type II (12/21 lesions; 57.1%), followed by type IV (4/21 lesions; 19%), type I (3/21 lesions; 14.3%), and type III (2/21 lesions; 9.5%).

**Table 1:** Table 1 General patient characteristics (n=18 patients and 21 venous malformations)

Characteristic	All patients
Gender (n=18)	
Male	7 (38.9%)
Female	11 (61.1%)
Location of venous malformations (n=21)	
Head and neck	8 (38.1%)
Upper extremities	2 (9.5%)
Lower extremities	9 (42.9%)
Trunk	2 (9.5%)
Puig’s classification of venous malformations (n=21)	
Type I	3 (14.3%)
Type II	12 (57.1%)
Type III	2 (9.5%)
Type IV	4 (19.0%)

Posttreatment, 8/21 lesions (38.1%) showed an excellent response in lesion size reduction, 9/21 (42.9%) showed a good response, and 3/21 (14.3%) showed an average response, while only 1 (4.8%) did not respond to treatment (**Table 2**). Fifteen lesions (types I and II) needed 28 injections to achieve a therapeutic effect, and 6 lesions (types III and IV) required 18 injections (**Table 3**). The pain score (according to VAS) at the 1-month, 3-month, and 6-month follow-ups were 1.45±1.3, 0.68±1.0, and 0.64±1.0, respectively, which were significantly improved as compared with that pretreatment.

**Table 2:** Table 2 Reduction in lesion size on magnetic resonance imaging in response to treatment (n=21 venous malformations)

Response level	Number of venous malformations (%)
Excellent	8 (38.1%)
Good	9 (42.9%)
Average	3 (14.3%)
No response	1 (4.8%)

**Table 3:** Table 3 Number of injections received according to the type of venous malformation (n=21)

Puig’s classification of venous malformations	Number of venous malformations	Number of injections
Types I and II	15	28
Types III and IV	6	18

Out of the 21 cases, 4 (19%) were associated with symptoms immediately after sclerotherapy, all of which involved chest tightness, while 2 cases had dry cough. No serious complications altering pulse, temperature, blood pressure, and respiration rate were noted. All patients posttreatment showed a nonserious local inflammatory response that appeared between day 1 and day 7. No other complications occurred posttreatment.

No recurrence was observed in the prospective group of 14 patients. In the retrospective group of four patients, recurrence occurred in three patients 1–4 years posttreatment, two of whom showed an increase in the lesion size on MRI. One patient had an increase in both the lesion size and pain score.

## Discussion

In this combined prospective and retrospective cohort study, we described the angiographic imaging characteristics of superficial venous malformations and evaluated the effectiveness of DSA-guided foam sclerotherapy with polidocanol for treating venous malformations. Our findings indicate that it is a safe and effective procedure for reducing the lesion size and pain in symptomatic patients with superficial venous malformation.

All included patients were mostly young with an average age of 26.5±12.9 years; females comprised ∼61.1% of the cohort and males ∼38.9%. This demographic is similar to that of previous studies such as Lee et al. (318 patients, 40.8% male and 59.2% female; average age, 24.9),^9)^ Su et al. (average age, 22.9 years),^10)^ and Li et al. (57% male and 43% female).^11)^

**Figure 1:**
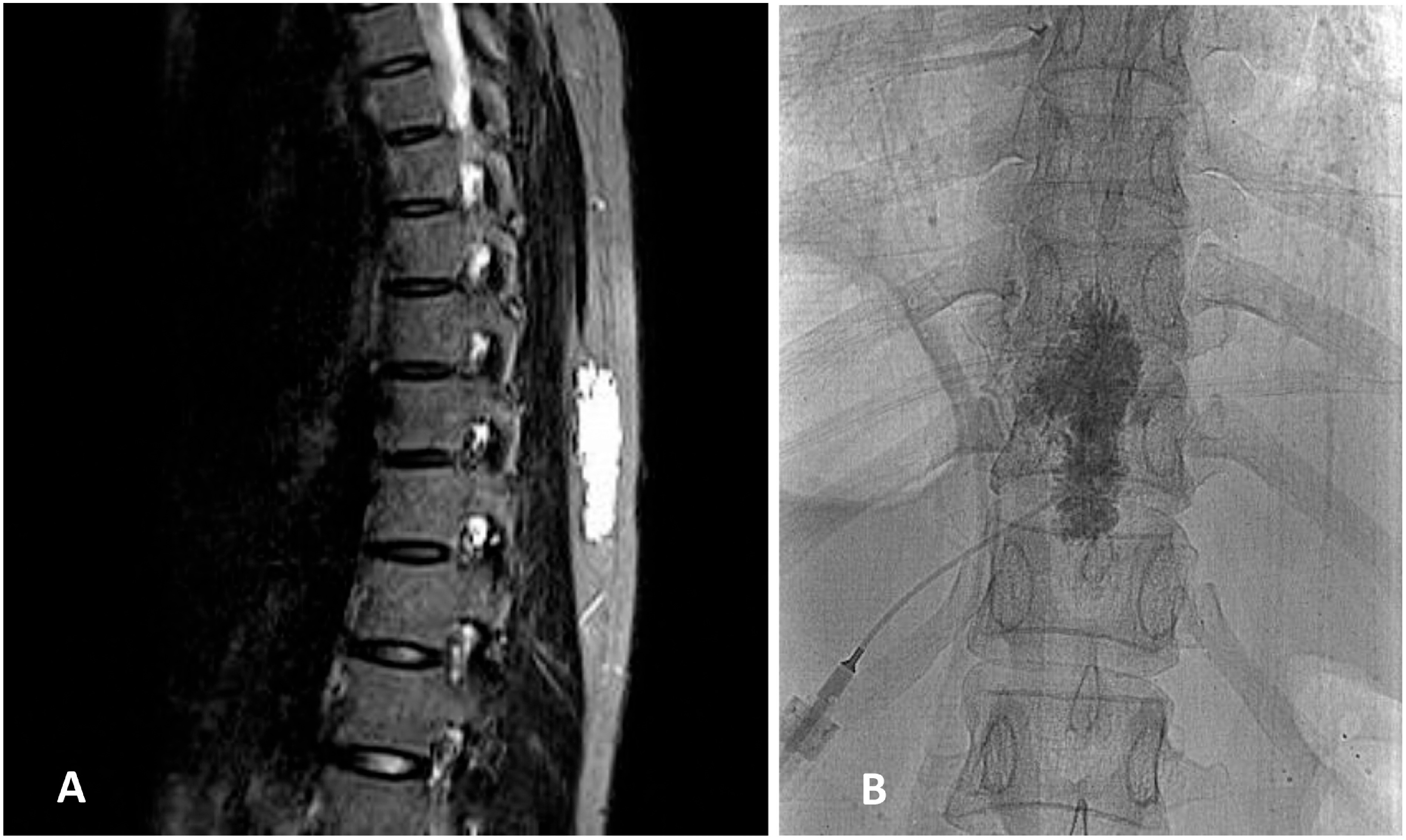
Fig. 1 A 26-year-old female patient experienced swelling and a painful mass in her back for many years. (**A**) MRI of the venous malformation. (**B**) DSA image shows a small drainage vein under the skin that did not drain into the medullary vein. This malformation was classed as type II according to Puig et al.^8)^

DSA: digital subtraction angiography; MRI: magnetic resonance imaging

**Figure 2:**
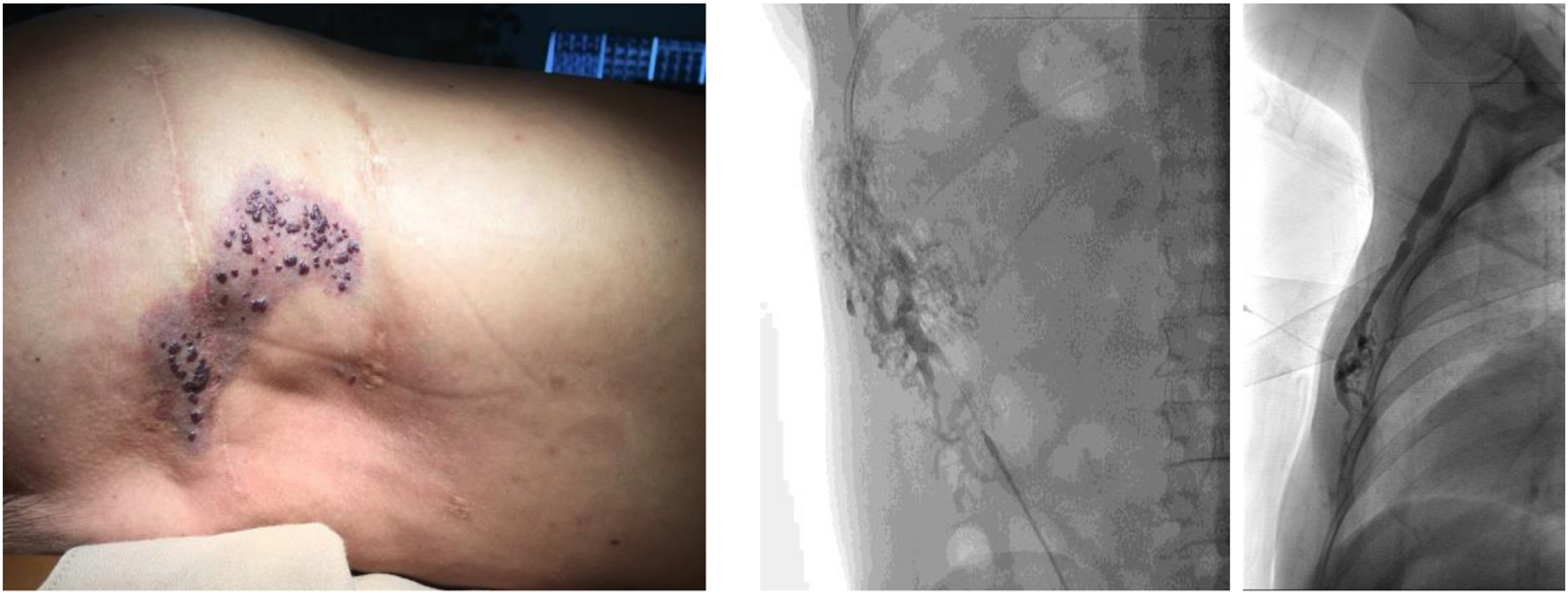
Fig. 2 A 25-year-old male with pain and a swelling mass in his back. This patient had received two previous surgeries over the past 5 years. DSA revealed a venous malformation with a drainage vein connecting to the axillary vein. The lesion was classified as type III according to Puig et al.^8)^ A second venous malformation contained ectasis veins and was classified as type IV.

We used Puig’s classification to divide venous malformations into four types. This classification is mainly based on the characteristics of the drainage veins. We found that type II was the most common (57.1%), followed by type IV (19%), type I (14.3%), and type III (9.5%), as shown in **Figs. 1** and **2**. This is consistent with the findings of the studies by Puig et al.^8)^ and Li et al.,^12)^ in which type II was the most common (37% and 53.3%, respectively).^12)^

DSA: digital subtraction angiography

The total number of injections required during the sclerotherapy for all patients in the present study was 46, with a mean of 2.19±1.7 injections per lesion (range, 1–7). The number of required injections in venous malformation types I and II was lower than that for types III and IV (p=0.03<0.05) with 95% confidence. This may be explained by the difference in drainage vein features according to the type of venous malformation. Types III and IV contain dysplastic drainage veins and ectasis veins; therefore, the flow through the drainage vein is often large, and the foam can easily pass through into the systemic circulation. In addition, the distribution of vessels in types III and IV malformations tends to be more pervasive than that in types I and II, making it difficult for the foam to spread, which explains why types III and IV required a greater number of injections.

We used MRI to evaluate the improvement in lesion size posttreatment because it has advantages over ultrasound such as optimal spatial and time resolutions, allowing a more accurate size assessment. To allow for complete resolution of inflammation post sclerotherapy, we reexamined via MRI at 6 months posttreatment.

Puig’s classification of venous malformations is well recognized and widely utilized because of its practical value.^4)^ It enables prognosis of the treatment response as well as potential complications.^13)^ However, the effectiveness of sclerotherapy remains controversial.^1,14)^ In the present study, the lesion size reduced posttreatment in 95.2% of cases; only one case, involving a venous malformation type IV on the back, failed to respond to treatment, and recurrence was recorded, with no significant response to sclerotherapy. In this particular case, we believe that previous surgical processes resulted in fibrosis scars, which separated the lesions into small parts that did not communicate with each other, making it difficult for the foam sclerosant to spread in the lesion. Furthermore, type IV has been previously shown to have a poorer response to treatment compared to types I and II.^8)^

All patients were admitted to our hospital due to complaints of pain. There was a significant improvement in the pain scores after 1 month, 3 months, 6 months, and >6 months posttreatment. The average pain score before and after treatment was 5.45 and 0.64, respectively, and this difference was statistically significant (two-tailed t-test; p=0.000, <0.01, with 99% confidence). These findings are consistent with other studies worldwide. Johnson et al. reported a significant improvement in swelling and pain in 85.7% of patients.^15)^ Mimura et al. showed a significant improvement in the pain score from a pretreatment average of 6.6–2.4 posttreatment.^16)^

In the present study, during the intervention period, four patients (19%) presented symptoms such as dry cough and chest tightness, which appeared immediately after termination of sclerotherapy foam pumping. However, these symptoms were mild and did not affect vital signs or self-healing. These symptoms are caused by reflux of foam sclerosant into the systemic circulation and occurred in type II or III cases (with drainage veins). We used Garo wire to close draining veins for the lesion in the extremities. With the lesions in the head or neck, we blocked the draining by hand. In our study, one of these patients had a venous malformation located under the left jaw, an area where it is difficult to block the drainage vein, containing a fast-flowing large drainage vein. Therefore, the foam sclerosant did not remain in the lesion; instead, it was refluxed into the systemic circulation through the drainage vein, resulting in complications and a lack of treatment response. The remaining lesions were located in the lower extremities and deep into the muscles. Therefore, we could not completely block the drainage vein using the foam, and a small flow persisted in the drainage vein. No serious complications were recorded after the intervention, consistent with the findings of previous studies worldwide.^17)^

The evaluation of recurrence after intervention was based on either an increase in pain scale or lesion size on MRI. We have 3 of the 18 patients (16.7%) who experienced recurrence symptoms after 1–4 years of follow-up, among which 2 patients showed an increased lesion size, and 1 patient showed both an increase in both lesion size and pain score. Posttreatment recurrence is believed to be a natural trend of the vascular malformation because vascular endothelial cells originate in the early stages of the fetal period; therefore, the cell growth characteristics of this period are retained. In addition, the insufficient spread of the foam sclerosant was also a factor that contributed to posttreatment recurrence.

Our study has several limitations, such as small sample size and short-term follow-up. Furthermore, all patients in our study were treated by sclerotherapy under DSA guidance. In other words, no cases were managed without using fluoroscopy to compare the results between the two techniques with or without using fluoroscopy. MRI is better, but it is difficult to evaluate the success during injection.

In conclusion, sclerotherapy under DSA guidance with polidocanol is a safe method to reduce the lesion size and pain in patients with superficial venous malformations. Further studies are strongly recommended in the field with a larger sample size and long-term follow-up to ascertain conclusions drawn from this study.
